# Target of initial sub-movement in multi-component arm-reaching strategy

**DOI:** 10.1038/s41598-019-56430-x

**Published:** 2019-12-27

**Authors:** Luka Peternel, Jan Babič

**Affiliations:** 1 Department of Cognitive Robotics, Delft University of Technology, Mekelweg 2, 2628CD Delft, The Netherlands; 2 Laboratory for Neuromechanics and Biorobotics, Department for Automation, Biocybernetics and Robotics, Jožef Stefan Institute, Jamova cesta 39, 1000 Ljubljana, Slovenia

**Keywords:** Motor control, Neurophysiology

## Abstract

Goal-directed human reaching often involves multi-component strategy with sub-movements. In general, the initial sub-movement is fast and less precise to bring the limb’s endpoint in the vicinity of the target as soon as possible. The final sub-movement then corrects the error accumulated during the previous sub-movement in order to reach the target. We investigate properties of a temporary target of the initial sub-movement. We hypothesise that the peak spatial dispersion of movement trajectories in the axis perpendicular to the movement is in front of the final reaching target, and that it indicates the temporary target of the initial sub-movement. The reasoning is that the dispersion accumulates, due to signal-dependent noise during the initial sub-movement, until the final corrective sub-movement is initiated, which then reduces the dispersion to successfully reach the actual target. We also hypothesise that the reaching movement distance and size of the actual target affect the properties of the temporary target of the initial sub-movement. The increased reaching movement distance increases the magnitude of peak dispersion and moves its location away from the actual target. On the other hand, the increased target size increases the magnitude of peak dispersion and moves its location closer to the actual target.

## Introduction

The understanding of underlying principles of target-directed movements can help us to improve the human rehabilitation and engineering (robotics in particular). Past studies have identified and proposed several models of how the human central neural system (CNS) is controlling the reaching movements of human limbs. Many of the models focused on speed-accuracy tradeoff in the reaching movement^1–6^, which follows Fitts’ law^7^. Due to the presence of signal-dependent noise in the commands that are sent from CNS to the motor units (muscles)^2,4,8,9^, the faster motion tends to be less accurate since it requires higher motor activation and therefore the neural noise is proportionally amplified. The CNS therefore tries to find an optimal speed for the required accuracy to reach a given target.

Other models focused on cost-benefit tradeoff in the reaching movement^10–14^. Such models help to prescribe the optimal muscular effort (i.e., cost related to the required energy), which is related to the speed of motion, with respect to the subjective value of getting the reward (i.e., reaching the target in a reasonable time). Recently, we proposed a novel model that is able to simultaneously account for both speed-accuracy and cost-benefit tradeoffs^15^.

While the speed-accuracy and cost-benefit tradeoffs provide fundamental strategies for optimising the movement itself, several studies focused on explaining the higher-level strategies of reaching. The existence of multi-component strategy in goal-directed human reaching movements has been known for more than a century^5^. In a pioneering work, Woodworth^16^ observed that the goal-directed movement of the limb can be separated into two distinct phases: the initial (primary) sub-movement that brings the limb’s endpoint in the vicinity of the target as a fast as possible, and the final (corrective) sub-movement that is responsible for correcting any error arising from the previous phase and successfully reaching the target. Researchers frequently studied the aspects of each sub-movement in terms of sensorimotor control. Initially, Woodworth^16^ hypothesised that the initial sub-movement is purely ballistic (feed-forward), while the final sub-movement relies on a sensory-feedback control. However, later studies showed that the initial sub-movement may not be purely ballistic but can also include some sensory feedback^17^.

An important research problem of multi-component reaching strategy is also the transition from the initial to the corrective sub-movement in the final stage. In this direction, one aspect of the transition was explored by the models proposed in^2,4^, which improved upon the original model of Woodworth^16^ by including stochastic properties of human motor control. Hence, using speed-accuracy tradeoff, the speed of the initial sub-movement is optimised with respect to accuracy in a way that the need for the final corrective sub-movement is minimised. Another aspect that often characterises the transition is the deceleration at the end of the initial sub-movement and subsequent acceleration at the beginning of the corrective sub-movement^17^.

The past studies provide a lot of insight into the nature of the initial sub-movement control strategy^17–19^, how the initial sub-movement is optimised with respect to final sub-movement^2,4,20^, how the transition is characterised through the changes in motion^17,20^, how spatial variability of endpoint in horizontal plane characterises the different phases of reaching movements^21^, and how other factors such as visual feedback^22–24^, age^25–27^ and practise^28^ affect the kinematics of sub-movements. However, the missing aspect is the understanding of how the target for the initial sub-movement is determined and how the conditions, such as reaching distance and target size, affect it.

**Figure 1:**
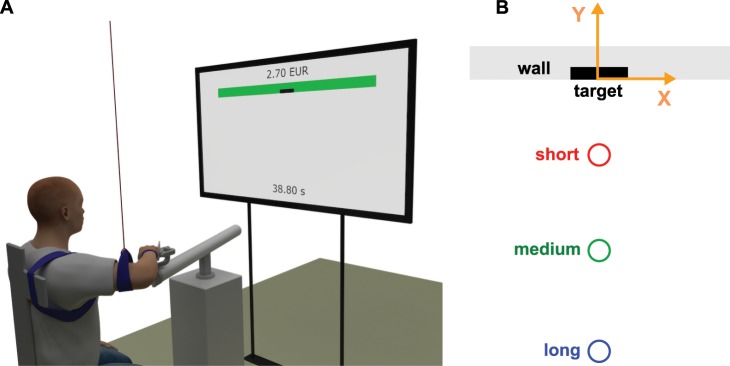
Experimental setup. (**A**) Illustration of subject performing experiments. The monitor in front of the subject displays the hand position in real time. In addition, the accumulated reward in euros and available remaining time in seconds are also displayed. (**B**) Target on a wall and different initial points marked with different colours. The reference frame is indicated by orange colour, where x-axis is perpendicular to the main movement direction and y-axis is parallel to the main movement direction.

In this paper, the main hypothesis is that the peak spatial dispersion of repeated movement trajectories in the axis perpendicular to the movement (i.e., along x-axis in Fig. 1B) is in front of the actual target (i.e., along negative y-axis in Fig. 1B), and that the peak dispersion indicates a temporary target, which serves as a reference for the initial sub-movement (*actual target*, or simply *target*, is the area where the limb has to reach to achieve the main goal, while *temporary target* is a temporary target that the hand has to reach at the end of the initial sub-movement). The underlying reasoning is that the error (i.e., dispersion of trajectories) accumulates due the signal-dependent noise during the initial sub-movement until the final corrective sub-movement is initiated, which then reduces the error to successfully reach the actual (final) target. Additionally, we hypothesise that reaching distance and actual target size affect the target of the initial sub-movement in the following manner:

- The increased reaching distance increases the size of the temporary target (characterised by the magnitude of peak dispersion) since the inaccuracy increases due to the faster movement being used to cover longer distance.
- The increased reaching distance moves the temporary target away from the actual target. To cover the longer distance, the faster movement accumulates higher inaccuracy during the initial sub-movement, therefore moving initial sub-movement target away from the actual target gives the final corrective sub-movement more space and time to reach the actual target.
- The increased actual target size increases the size of the temporary target since larger actual target permits more inaccuracy during the initial sub-movement.
- The increased actual target size moves the temporary target closer to the actual target since larger actual target permits less complex final corrective sub-movement.

## Materials and Methods

To test the proposed hypotheses we conducted an experimental study where a human subject had to reach a target with the hand using arm movement (see Fig. 1A for illustration). The experimental setup included three starting points located along the line perpendicular to the wall at different distances from the target (i.e., 15%, 37.5% and 60% of the arm length (see Fig. 1B for illustration)). The main experiment was divided into four sessions, each corresponding to a different target size (5 mm, 10 mm, 20 mm and 40 mm). The sequence of sessions was selected randomly for each subject. Before the main experiment, each subject had to do a preliminary session with 20 mm target size for a familiarisation purpose in order to minimise effect of learning on the main experiment. In each session, the initial points varied randomly between the given three distances from the target.

The subjects were given a reward of 2.5 euro cents if they successfully reached the target and no reward if they missed the target. In each session, they could gather rewards through reaching movements only for a limited amount of time in order to be compelled to accumulate the highest possible final reward. After each movement (trial), the time of the movement was deducted from the total time available in the session. Time of movement was defined from the moment the subject exited the initial point and to the moment the wall was touched (whether the target was hit or missed). The time necessary for moving back to the initial points was not counted. The time the subject prepared for the movement inside the initial point was also not counted. When the time ran out, the session was finished and the subject could not get more reward. Essentially, the less time the subjects spent on individual movements, the more they could make them in the total available time and the more reward they could potentially accumulate (if the target was successfully hit) before the time ran out. This ensured that the subjects followed the speed-accuracy and cost-benefit tradeoffs^15^. The number of movements (trials) in each session therefore varied and depended on the subject’s performance. Individual movement times similarly varied for the same reason.

We used a TV monitor in front of the subject to display the starting points, the target and real-time position of the hand (see Fig. 1A for illustration). The actual hand position was not visually obstructed. In addition, reach or miss information was indicated after each movement, while the cumulative reward and remaining time were displayed at the top and at the bottom of the screen, respectively. The TV monitor was positioned two meters from the backrest of the chair where the subject was sitting. We fixed the subject’s shoulder position by tying the trunk of the subject to the backrest of the chair. We measured the reaching movements with a dedicated haptic manipulator (Haptic Master Mk2, MOOG, Nieuw-Vennep, The Netherlands). The subject’s right hand was connected to a haptic manipulator and the wrist joint was immobilised through the gimbal mechanism (ADL gimbal mechanism, MOOG, Nieuw-Vennep, The Netherlands). The motion of the hand was constrained to the horizontal plane at the height of the subject’s shoulders by the haptic manipulator. Furthermore, to restrain the arm motion to the horizontal plane and to compensate for its gravity, we suspended the right elbow of the subject from the ceiling by a long string.

Before each movement, the subject had to keep the hand within a circular area of the initial point with diameter of 10 mm. The movement toward the target was initiated whenever the subject moved out of the area of the initial point. The target was was located on a haptic wall generated by the haptic manipulator. The wall was aligned along the x-axis of the measurement reference frame (see Fig. 1B), which was parallel to the coronal plane of human body and perpendicular to the main movement direction. Since we were primarily interested in trajectory dispersion along x-axis, the subjects were not instructed to stop precisely at the target and could use the wall to help them stop the movement in the axis perpendicular to the target area, i.e., y-axis of the measurement reference frame (see Fig. 1B). Sampling rate was 100 Hz for the movement data, as measured by the haptic manipulator. The end of the movement was defined when the target surface (i.e., wall) was reached. We also recorded muscular activity through surface electromyography at 1000 Hz. To amplify the activation signals to measurable levels, the haptic manipulator emulated slightly viscous medium.

Ten healthy male subjects participated in the study, with an average age of 23.0 years (SD = 2.7 years), average height of 178.8 cm (SD = 4.1 cm) and average body mass of 77.4 kg (SD = 5.8 kg). The subjects were all right-handed and exclusion criteria included visual, vestibular, neurological and locomotion disorders or any recent limb injuries (self-reported). All experimental protocols were approved by the National Medical Ethics Committee Slovenia (NO. 112/06/13) and all research was performed in accordance with relevant guidelines and regulations. All subjects gave a written informed consent prior to their participation.

Statistical analyses were conducted in accordance with standard and established methods^29^ and were performed using JASP software^30^. For each subject, the average magnitude of the peak spatial dispersion of trajectories in x-axis (from now on: *magnitude of peak dispersion*) and its location on y-axis (from now on: *location of peak dispersion*) were determined for every combination of target size and reaching distance to the target (from now on: *target distance*. The average values of the individual subjects were then used for statistical analysis. Effects of target size and target distance on magnitude of peak dispersion and location of peak dispersion were determined by two-way repeated-measures ANOVA (Target size (4) × Target distance (3)). Differences between different target sizes and target distances were tested with post hoc t-tests with Bonferroni correction. The level of statistical significance was set to 0.05. We carried out a pilot study (n = 4), calculated power related parameters (alpha = 0.05, power = 0.80) and based on that a sample of 9 subjects or more turned out to be sufficient. Pilot study was conducted prior to the main study. Only the data from the main study was used in the results.

## Results

For 5 mm target, a subject performed on average 4.6 ± 0.9 trajectories in long, 4.9 ± 1.1 in medium and 5.2 ± 1.0 in short distance condition. For 10 mm target, a subject performed on average 5.3 ± 1.1 trajectories in long, 6.0 ± 1.2 in medium and 5.8 ± 1.2 in short distance condition. For 20 mm target, a subject performed on average 7.9 ± 1.2 trajectories in long, 7.8 ± 1.2 in medium and 7.9 ± 1.0 in short distance condition. For 40 mm target, a subject performed on average 9.9 ± 0.9 trajectories in long, 9.5 ± 1.4 in medium and 10.2 ± 1.2 in short distance condition.

### Magnitude of peak dispersion

**Figure 2:**
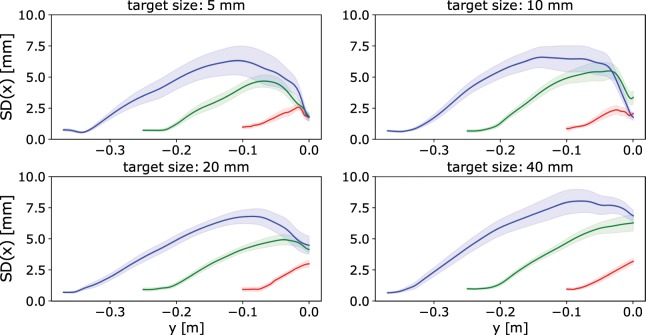
Dispersion of trajectories along the axis perpendicular to the main movement direction (x-axis) as a function of axis parallel to the main movement direction (y-axis). It was obtained as a mean dispersion of trajectories among individual subjects for each sample point, where the dispersion was represented by standard deviation. The solid line represents the mean value, while the shaded area represents the standard error of mean. Each graph corresponds to the different target size. Different colours correspond to the different target distances: red is short, green is medium and blue is long distance.

Figure 2 shows the dispersion of reaching trajectories in x-axis (perpendicular to the main movement direction) performed by the subjects during the experiments. For supplementary results regarding spatial trajectories and velocity profiles, please refer to Fig. 5 in Appendix A and Fig. 6 in Appendix B, respectively.

**Figure 3:**
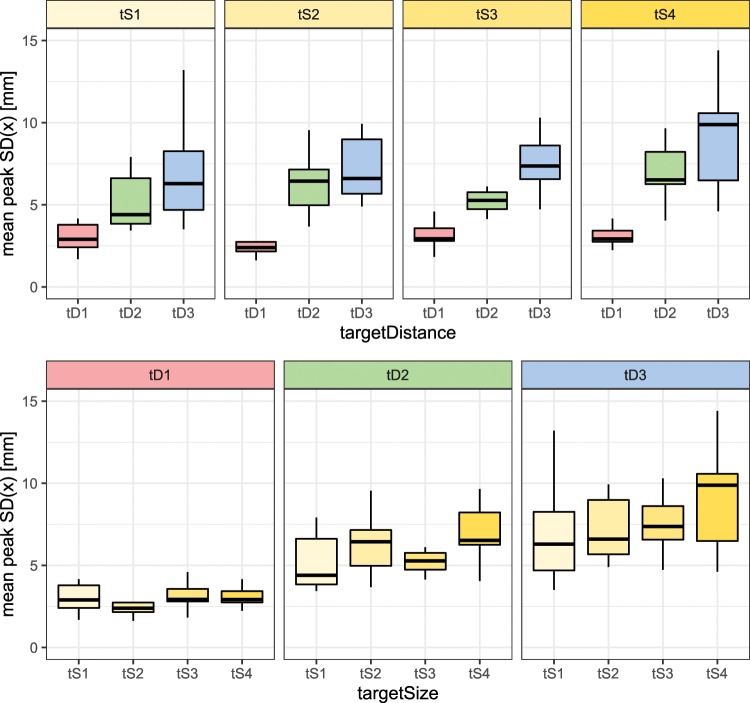
Maximum standard deviation of trajectories along the axis perpendicular to the main movement direction (x-axis). The box-whisker plots depict median (horizontal line inside the box), interquartile range (box) and extreme values (whisker) for a given variable. The top graphs are organised by different target size (tS1 is the smallest and tS4 is the largest). The bottom graphs are organised by different target distances (tD1 is the shortest and tD3 is longest). Note that the values in this graph are the mean of individual dispersion peaks of each subject, while the values in Fig. 2 are the mean of the dispersion trajectories themselves, therefore there is a difference between the mean of individual dispersion peaks in Fig. 3 and the peak of the overall mean dispersion in Fig. 2.

The dispersion of trajectories has a pronounced peak just before the target. The magnitude of peak dispersion changes considerably with the target distance (see the top graphs in Fig. 3). The increase of magnitude of peak dispersion with respect to the target distance is statistically significant ($F(1.30,11.68)=25.11$, $p < 0.001$, ${\eta }^{2}=0.74$). There was a significant linear trend ($t=10.90$, $p < 0.001$) indicating that as the target distance increased, the magnitude of peak dispersion increased proportionately.

The target size also affects the magnitude of peak dispersion (see the bottom graphs in Fig. 3). The change of magnitude of peak dispersion with respect to the target size is statistically significant ($F(1.71,15.36)=4.06$, $p=0.044$, ${\eta }^{2}=0.31$). While the pattern is not as clear and pronounced as for the target distance, there was a significant linear trend ($t=2.30$, $p=0.023$) indicating that as the target size increased, the magnitude of peak dispersion increased proportionately.

### Location of peak dispersion

**Figure 4:**
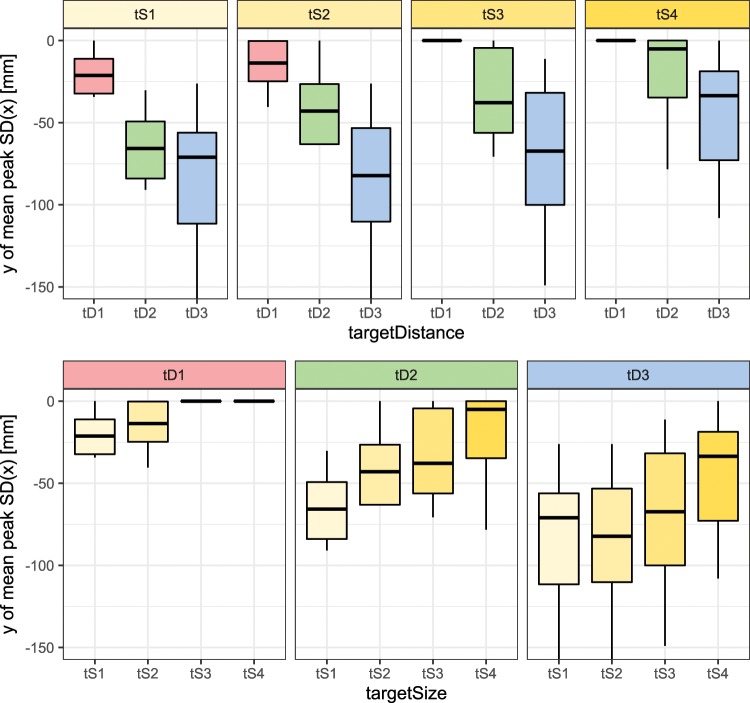
Location of maximum standard deviation of trajectories along the axis perpendicular to the main movement direction (x-axis) with respect to the axis parallel to the main movement direction (y-axis). The box-whisker plots depict median (horizontal line inside the box), interquartile range (box) and extreme values (whisker) for a given variable. The top graphs are organised by different target size (tS1 is the smallest and tS4 is the largest). The bottom graphs are organised by different target distances (tD1 is the shortest and tD3 is longest). Note that the values in this graph are the mean of individual peak y-axis positions of each subject, while the values in Fig. 2 are mean of the dispersion trajectories themselves, therefore there is a difference between the mean of individual locations of peak dispersion in Fig. 4 and the location of peak on the overall mean dispersion in Fig. 2.

The location of peak dispersion is strongly affected by the distance of reaching movement in a way that the peak moves away from the target with the increased distance (see the top graphs in Fig. 4). The change of location of peak dispersion with respect to the target distance is statistically significant ($F(1.67,15.02)=31.99$, $p < 0.001$, ${\eta }^{2}=0.78$). There was a significant linear trend ($t=-\,7.97$, $p < 0.001$) indicating that as the target distance increased, the location of peak dispersion moved away from the actual target proportionately.

However, similarly to the magnitude of peak dispersion, the target size affects the location of the peak in y-axis (along the main movement direction) much less compared to the target distance (see the bottom graphs in Fig. 4). The change of peak location with respect to the target size is statistically significant ($F(1.95,17.58)=3.81$, $p=0.043$, ${\eta }^{2}=0.30$). While the pattern is not as clear and pronounced as for the target distance, there was a significant linear trend ($t=4.28$, $p < 0.001$) indicating that as the target size increased, the location of peak dispersion moved towards the actual target proportionately.

### Movement times

Average movement times for the initial sub-movements are shown in Table 1. In this case, the time was measured from the onset of individual movement to the moment it reached peak dispersion. Average movement times for the final sub-movements are shown in Table 2. In this case, the time was measured from moment an individual movement reached its peak dispersion to the moment when the subject touched the wall, whether the target was hit or not.

**Table 1:** Initial sub-movement times in seconds (mean and SD) with respect to target distance and target size.

	tS1	tS2	tS3	tS4
tD1	0.59 ± 0.33	0.43 ± 0.19	0.39 ± 0.17	0.33 ± 0.11
tD2	0.94 ± 0.25	0.82 ± 0.20	0.57 ± 0.13	0.52 ± 0.09
tD3	0.88 ± 0.30	0.83 ± 0.28	0.88 ± 0.21	0.76 ± 0.19

**Table 2:** Final sub-movement times in seconds (mean and SD) with respect to target distance and target size.

	tS1	tS2	tS3	tS4
tD1	0.81 ± 0.34	0.72 ± 0.29	0.42 ± 0.22	0.41 ± 0.12
tD2	0.83 ± 0.29	0.77 ± 0.52	0.61 ± 0.18	0.56 ± 0.19
tD3	1.07 ± 0.43	0.96 ± 0.36	0.56 ± 0.17	0.47 ± 0.10

## Discussion

As indicated by the results in Fig. 2, the dispersion of trajectories in the axis perpendicular to the main movement direction (i.e., parallel to the target area) has a pronounced peak that is located eccentrically closer to the target. The dispersion arises from the signal-dependent noise that affects the accuracy of motor units^2,4,8,9^. As the trajectory goes further from the initial point of reaching, the error accumulates and the spatial dispersion increases. However, as observed in the experimental data, the peak dispersion is generally not located at the target but at some distance from the target. This implies that a final corrective sub-movement with a feedback control strategy is initiated after the peak dispersion in order to reduce the error and successfully reach the target. Hence, after the corrective movement, the dispersion drops as a result of reducing error with respect to the actual target. In relation to the main hypothesis, based on this we believe that the temporary target for the initial sub-movement is located at the peak dispersion, before the final corrective sub-movement is executed.

Several past studies, such as^21,22,31–33^, similarly observed that spatial dispersion increases in the initial phase and that it reduces in the final phase of the goal-directed reaching movements. However, they did not study the temporary target of the initial sub-movement, nor did they make any hypothesis or implications about it.

A study in^28^ observed that the spatial dispersion reduces with practise. Note that Elliott *et al*.^28^ examined the dispersion in the axis along the main movement direction (i.e., y-axis) in terms of undershoots and overshoots, while our study focuses on dispersion in the axis perpendicular to the main movement direction (i.e., x-axis). We performed a preliminary session before the main experiment for the familiarisation purpose in order to minimise the effect of practise/learning on the results.

According to the experimental results, the target distance has a dominant effect on the magnitude and location of peak dispersion. The longer the target distance is, the faster should the movement be in order to account for the cost-benefit tradeoff^10,11,15^. On the other hand, due to the inaccuracy induced by the signal-dependent noise, the faster movements become more imprecise. For this reason, the magnitude of peak dispersion (see Fig. 2) is higher for the longer target distances, compared to the shorter target distances. This suggests that the temporary target for the initial sub-movement is larger for longer target distances than for shorter target distances.

The temporary target moves away from the actual target as the reaching distance increases. This implies that CNS compensates for a more demanding corrective action, which arises from a higher accumulated inaccuracy during the initial sub-movement, by giving the final sub-movement more space and time to reach the actual target.

This is in line with the notion that higher error after the initial sub-movement requires more complex final corrective sub-movement^4,20^. Note however that in the past studies^4,20^ the target area was aligned with the main movement direction and they observed the dispersion in the axis of the main movement direction (i.e., undershoots and overshoots). On the other hand, in our study the target area was perpendicular to the main movement direction and we observed the dispersion in the axis perpendicular to the main movement direction.

According to the experimental results, the actual target size affects the magnitude and location of peak dispersion much less compared to the target distance. Nevertheless, as the target size increases the magnitude of peak dispersion slightly increases. Since larger actual target is easier to reach with the final corrective sub-movement, the temporary target of the initial sub-movement can also be larger.

In addition, as the target size increases, the location of peak dispersion moves slightly closer to the target. Since larger actual target is easier to reach and therefore less room and time is required for the final corrective sub-movement, the temporary target of the initial sub-movement can move closer.

As seen from the results, the split between the two movement components does not involve stopping at the virtual target (see velocity profiles in Fig. 6 in Appendix B of supplementary results). The split is characterised by the change of control strategy after the temporary target of initial sub-movement, i.e., switching from primarily feed-forward control to corrective feedback control. The feedback control does not stop the movement but rather changes the direction of movement toward the final target. We believe that there is no reason to stop at the temporary target, unless when some extreme anomaly in the primarily movement occurs or when the final target is extremely small.

For the shortest target distance, the time of the initial sub-movement is generally lower compared to the time of the final sub-movement (see Tables 1 and 2), while for the medium and long distances, the time of sub-movements is generally comparable. That is despite the initial movement covering a larger distance, because the initial sub-movement has much higher average speed compared to the final sub-movement (see also velocity profiles in Fig. 6 in Appendix B of supplementary results). The time of both sub-movements generally decreases with the increased target size. This is because larger target is easier to hit and therefore the speed can be increased in accordance to the speed-accuracy tradeoff.

Movement references, such as temporary targets of sub-movements, are primarily higher-level parameters created within CNS. Like many other human motor control studies^2,4,8–10,20,34–36^, we studied the function of CNS indirectly through movement observation. Without full access to the cognitive process of CNS, observing actual body movements can mostly provide circumstantial evidence. However, the results of this study, in combination with established principles of motor control from the state-of-the-art literature, provide a strong evidence that the temporary target of the initial sub-movement is located at peak spatial dispersion in the axis perpendicular to the movement (and parallel to the actual target). In line with well-established signal-depended noise principle of motor control^37^, the spatial dispersion will increase in the ballistic sub-movement, unless the corrective sub-movement with feedback strategy is initiated. Therefore, the dispersion should be at maximum just before the corrective movement is applied.

It is also important to discuss the particular conditions with respect to the type of the final target, which might affect the variability in different axes of reference frame and therefore temporary target of the initial sub-movement. In this study, the target was flat and oriented perpendicularly to the main movement direction, therefore the temporary target would be oriented in a similar manner to best prepare for hitting the final target. Hence the spatial dispersion is most important in the axis perpendicular to the main movement. However, if the final target has different shape/properties (e.g., point target, round target, etc.), then other axes might become more prominent^23^. Note that variability in the axis of the main movement direction can be inferred from Fig. 4.

In addition, in this study the target was solid (on the wall) and the human could use it to help stop the movement at the end (this corresponds to various daily-life tasks, such reaching for a solid handle on a door/window, etc.). However, if the target is delicate and the human has to stop precisely on the target, the spatial dispersion in the axis of the main movement direction becomes more important. In such case for example, undershoots and overshoots become prominent factor in the reaching movement^20,28,38^. While many studies in the literature investigated delicate type of targets, not much research is available on solid type of targets; therefore we were motivated to study the latter. Another reason to choose a solid target on a wall was that it is much easier to determine the hit dispersion and end of the movement (i.e., movement time). If the target can be overshoot, the hit dispersion and end of the movement are more ambiguous.

Finally, it should be acknowledged that some motor control views, studies and models suggest that humans use single-component reaching movement strategy or do not explicitly mention/incorporate multi-component strategy (e.g.^9–11,34–36,39^). However, the strategy might depend on different conditions, such as: length and difficulty of movement, type of target, cost of failure, etc. According to the results in Figs. 2 and 4, decrease of target distance and increase of target size move the dispersion peak very close to or directly on the actual target, which may imply that multi-component strategy is not necessary for shorter movements with larger targets.

The results of this study provide novel insights into the target of the initial sub-movement of goal-directed reaching and can potentially be useful for designing new computational models of motor control in human neuroscience and robotics.

## Supplementary information

Supplementary Data 1
