# Supplementary material for: Target of initial sub-movement in multi-component arm-reaching strategy
Source: Sci Rep. 2019 Dec 27;9:20101. doi: 10.1038/s41598-019-56430-x (PMC6934494; doi:10.1038/s41598-019-56430-x)
Supplement: Supplementary file 1 — Supplementary Data 1 [file 41598_2019_56430_MOESM1_ESM.pdf]

Target of initial sub-movement in multi-component arm-reaching strategy

Author Names and Affiliations: Luka Peternel[1*] and Jan Babič[2]

[1]: Department of Cognitive Robotics, Delft University of Technology, Mekelweg 2, 2628CD Delft, The Netherlands
[2]: Laboratory for Neuromechanics and Biorobotics, Department for Automation, Biocybernetics and Robotics, Jožef Stefan Institute, Jamova cesta 39, 1000 Ljubljana, Slovenia
[*]: Corresponding Author: l.peternel@tudelft.nl

Appendices

A Movement trajectories

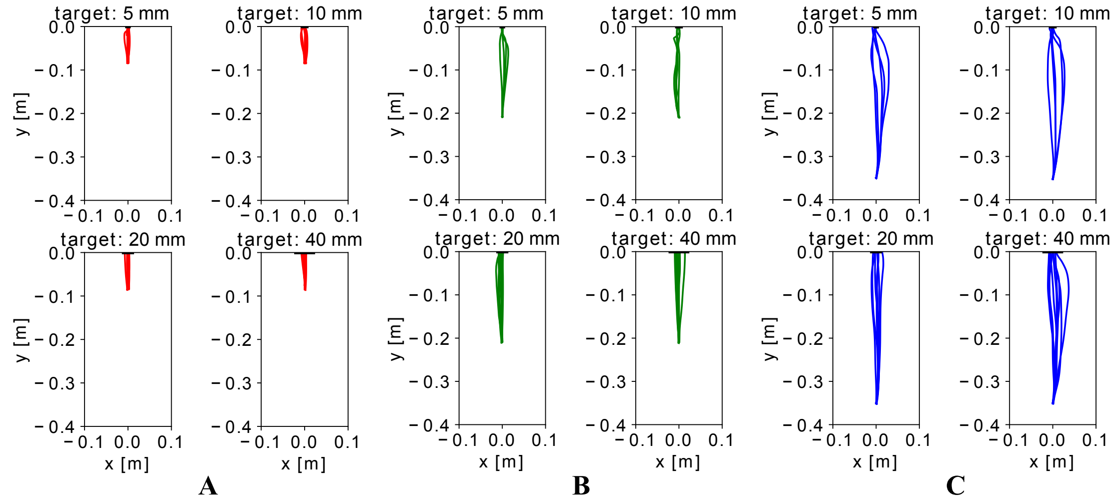

A B C

Figure 5: Examples of movement trajectories from the initial point to the target/wall in different conditions from different subjects. Each graph corresponds to the different target size. Each block of graphs is marked with different colours and corresponds to different target distance (A is short, B is medium and C is long).

B Velocity profiles

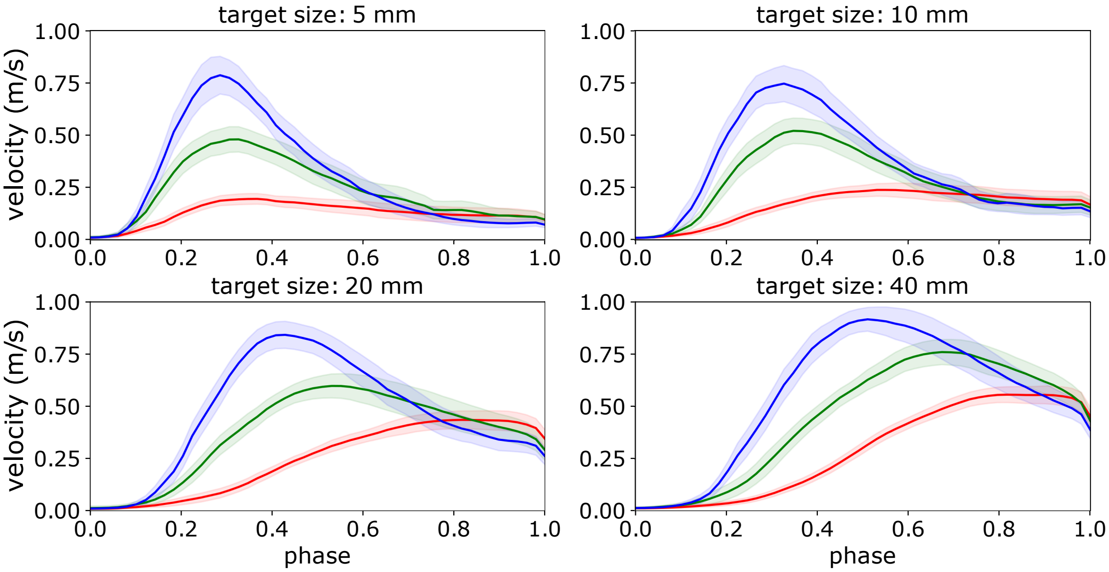

Figure 6: Velocity of movement with respect to phase (i.e., normalised time). The solid line represents the mean value, while the shaded area represents the standard error of mean. Each graph corresponds to the different target size. Different colours correspond to the different target distances: red is short, green is medium and blue is long distance.
